# Efficacy of rTMS for poststroke epilepsy and its effects on patients’ cognitive function and depressive status

**DOI:** 10.1186/s12883-024-03531-4

**Published:** 2024-01-12

**Authors:** Minting Hu, Bailing Qin, Tong Li, Chunyan Wei, Dajing Su, Zuocai Tan

**Affiliations:** Department of Neurology, The Second People’s Hospital of Nanning, Nanning, China

**Keywords:** Repetitive transcranial magnetic stimulation (rTMS), Poststroke epilepsy, Cognitive impairment, Depression, MMSE, HAMD

## Abstract

**Objective:**

This study aimed to investigate the efficacy of rTMS in the treatment of poststroke epilepsy and the effect of rTMS on patients’ cognitive function and depressive status.

**Methods:**

One hundred and twenty-one poststroke epilepsy patients with mild cognitive impairment and depressive status admitted to the Department of Neurology of the Second People’s Hospital of Nanning from January 1, 2017, to April 31, 2023, were selected and divided into the rTMS treatment group (treated group) and the control group. MMSE scores and HAMD scores were recorded before and after treatment. The frequency of EEG spiky waves recorded before and after treatment within 24 h and the frequency of any clinical seizure form (the number of clinical seizures within 1 month after treatment) and changes in observed indices before and after treatment were calculated. The differences between the data of the two groups were analyzed, to further assess the efficacy of rTMS in the treatment of poststroke epilepsy and the rTMS’ effects on cognition and depression.

**Results:**

Compared with drug treatment alone, rTMS significantly decreased clinical seizures and epileptiform discharges after stroke, especially in patients with lesions in the frontal, temporal, and parietal lobes. Compared with drug treatment alone, rTMS treatment can effectively reduce cognitive impairment and mood disorders, such as depression, especially for patients with lesions in the frontal and temporal lobes. The results of this experiment suggest that rTMS treatment does not increase adverse effects.

**Conclusion:**

rTMS reduces clinical seizures while improving cognitive impairment and depression in patients with epilepsy. Therefore, we suggest that low-frequency rTMS can be used as an adjunctive treatment for patients with epilepsy and provide some ideas and references for the treatment of epilepsy with cognitive impairment and depression.

**Supplementary Information:**

The online version contains supplementary material available at 10.1186/s12883-024-03531-4.

## Introduction

Poststroke epilepsy often occurs in stroke patients [1]. Epilepsy often is associated with cognitive impairment and depression, which have a common pathological process and may occur simultaneously or even contribute to each other. Moreover, the longer the duration of epilepsy, the more severe the cognitive impairment and depression [2]. Cognitive impairment is mild in patients with an early diagnosis of epilepsy, therefore may go unnoticed and persist for long periods [3]. Similarly, depression in poststroke epilepsy patients is often not recognized, and patients often refuse treatment for fear of stigmatization [4]. Depression can cause not only many emotional and social problems [5], but also cognitive impairment [6]. Therefore, the treatment of cognitive impairment and depression associated with poststroke epilepsy needs to be established soon. On the other hand, some antiepileptic drugs have been clinically shown to cause depressive symptoms or intellectual disability [5]. Conversely, some antidepressants may also affect epilepsy control [7]. Therefore, the ideal treatment for patients with epilepsy should be a focal treatment that does not cause an increase in clinical seizures but reduces cognitive impairment and depression.

rTMS is a form of brain stimulation based on a time-varying magnetic field—A small coil with a strong and fast alternating current is applied to the cerebral cortex [8, 9]. It can reduce clinical seizures [10, 11], and be beneficial in early treatment for the improvement of intellectual and emotional disturbances [2, 12], too.

At present, researches on the treatment of stroke patients with rTMS mostly focus on the recovery of Limb Motor Function [13–15], and some studies focus on stroke patients’ cognitive impairment [15, 16] and depression [17, 18], with most of the therapeutic effects being positive and effective. However, no studies have evaluated the effects of low-frequency rTMS on combined cognitive impairment and depression in patients with poststroke epilepsy, and it cannot be determined whether rTMS can reduce clinical seizures while leading to improvements in cognitive function and mood disturbances. Therefore, we boldly assume that rTMS can improve patients’ cognitive impairment and depression by reducing post-stroke seizures, and carry out this experimental research.

## Materials and methods

### Experimental subjects

One hundred and twenty-one patients with poststroke epilepsy with cognitive impairment and depressive status attending the Department of Neurology in our hospital from January 1, 2017 to April 31, 2023 were selected for the study, and all patients were treated with standardized secondary stroke prevention medication according to the Chinese Guidelines for the Diagnosis and Treatment of Acute Ischemic Stroke 2018 [19] and antiepileptic drugs (levetiracetam tablets, Zhejiang Huahai Pharmaceutical Co., Ltd, State Drug Administration H20203042, specification: 500 mg, twice daily (1 g each time).

This study was a prospective randomized controlled clinical trial. Those patients were subdivided into control and treated groups using a computer-generated list of random numbers. The conventional drug group was the control group, and the treated group was treated with levetiracetam and 0.5 Hz continuous treatment for 1 week.

To determine the sample size, we assumed a mean reduction in seizures of at least 75% in the treated group. In the control group, we assumed a mean reduction of seizure frequency of 50%. After consultation with researchers and statisticians, we considered the power of 70% and the critical α = 0.05 (double-sided), so that 92 patients (46 in each group) are needed to detect group differences.

#### Inclusion criteria

- Greater than 18 years of age.
- Compliance with the diagnostic criteria for cerebral infarction in the Chinese Guidelines for the Diagnosis and Treatment of Acute Ischemic Stroke 2018 [19].
- Meeting the diagnostic criteria for poststroke epilepsy in the Chinese expert consensus on clinical diagnoses and treatments of poststroke epilepsy [20]. Two or more unprovoked clinical seizures or EEG-confirmed epileptiform discharges largely consistent with the stroke site after stroke.
- The course of drug treatment conforms to the treatment specifications of the Chinese Expert Consensus on the diagnosis and treatment of poststroke epilepsy [20].
- The patient or his legal representative signs the informed consent form.
- Diagnosis of mild cognitive impairment (mini-mental state examination (MMSE) score of 21 to 26).
- Diagnosis of having a depressive state (Hamilton Depression Scale (HAMD) score ≥ 8).

#### Exclusion criteria

- History of epilepsy or depression before stroke.
- The presence of other diseases that can cause convulsions (e.g., central nervous system diseases (intracranial infections, subarachnoid hemorrhage, cerebral arteriovenous malformations, cavernous hemangiomas, cortical venous infarcts, impaired global cognitive functioning, etc.) or systemic diseases (blood glucose abnormalities, electrolyte disturbances, intoxication, fever, etc.));
- Communication barriers that prevent the participants from understanding the process;
- Contraindications related to rTMS, such as the presence of metallic magnetic materials in the skull or body cavity (e.g., stents, cochlear implants, some pacemakers, and other implantable medical products);
- Family history and medical history of mental disorders;
- Taking concomitant drugs that alter cortical excitability (e.g., antidepressants, neurological stimulants, and psychoactive depressants);
- History of drug or alcohol abuse with withdrawal syndrome;
- Subarachnoid hemorrhage and postinfarction hemorrhagic transformation during treatment;
- Patients who do not sign the informed consent form or who are unable to cooperate and participate in the whole trial and withdraw in the middle of the trial.

### Methods

#### Data collection

The following baseline data were collected by a neurologist at the time of the patient’s visit: (1) demographic characteristics: sex, age, genetic factors (family history of epilepsy), etc.; (2) vascular risk factors: hypertension, hyperlipidemia, diabetes mellitus, coronary artery disease, atrial fibrillation, smoking (≥ 6 months, ≥ 10 cigarettes/d), and alcohol consumption (≥ 6 months, > 30 g/d or 210 g/week); and (3) medication use.

**Fig. 1:**
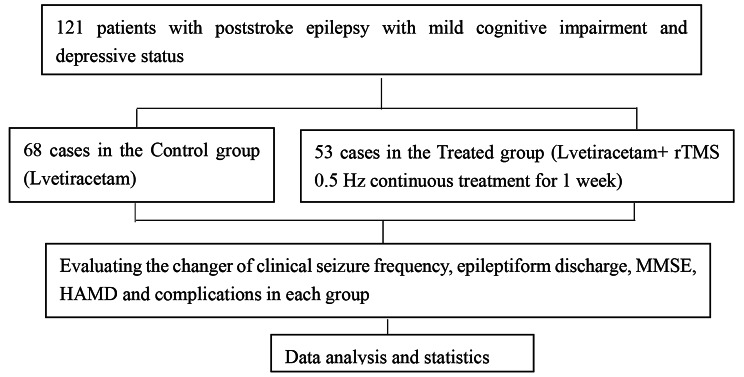
Experimental process

MMSE scores and HAMD scores were recorded before and after treatment; the frequency of EEG spiky waves was recorded in the 24 h before and after treatment, as well as the frequency of any form of clinical seizures (number of clinical seizures within 1 month after treatment); the degree of improvement in clinical seizures and their treatment efficiency, the degree of improvement in EEG and their treatment efficiency, the degree of improvement in MMSE, the degree of improvement in HAMD and their treatment efficiency (Fig. 1).

#### Imaging and brain electrophysiology equipment

All patients in the two groups completed cranial CT + CTA or MRI scan + MRA (angiography), MRI sequences with 3D-T1WI, T2WI, FLAIR, DWI sequences, CT or MRI examination to understand the imaging changes of vascular lesions and infarct sites and to exclude other diseases. All patients in the two groups were required to undergo EEG, and the EEG was reexamined after 1 month of intervention, and to check the Therapeutical Drug Monitoring (TDM) of Lvetiracetam weekly.

### (1) Imaging technology

MRI scans were performed using SIEMENS 3.0T high-field MRI. A GE Gemstone Energy Spectrum CT (Discovery CT 750 HD) was used for the cranial CT scan.

### (2) Brain electrophysiological examination

Lectrode locations are based on the international 10–20 system, using the same earlobe referential montage, with a recording time of 3 h.There are a total of 21 electrodes. EEG data were acquired using a NeuronSpectrum-5 digital neurophysiology system manufactured by Neurosoft Llc.

### (3) rTMS treatment modality

The resting motor threshold (rMT) of the patient’s cortical layer was measured at the first treatment using a YD-MT500 Magnetic Stimulation Therapy System manufactured by Henan Youde Medical Equipment Co. The patient was placed in a sitting or supine position, and the thumb motor area (M1) on the lateral side of the hand (Chinese are mostly right-handed) was stimulated in single-pulse mode. They underwent stimulation 10 times, of which 5 times could induce thumb abductor movements (inducing thumb abductor evoked potentials of 50 micro amplitudes or more), and the intensity of this stimulation was rMT. ②Target localization of rTMS treatment: The area of epileptiform discharges is the stimulation area. Localization with reference to the international standard EEG electrode 10–20 lead system. ③The 8-word coil was selected, and 0.5 Hz low-frequency intervention was given with 90% resting motor threshold (rMT) stimulation intensity, 1200 pulses/day were continuously repeated, lasting about 40 min. The treatment course was 1 week.

#### Clinical efficacy determination criteria (primary outcome)

More than a 50% reduction in clinical seizure frequency compared with that before treatment was effective, and less than 50% was ineffective. The productivity of seizure frequency (%) = the number of effective cases/the total number of cases × 100%. The degree of change in seizure frequency values before and after treatment (%) = (the frequency of clinical seizures before treatment - the frequency of clinical seizures after treatment)/the frequency of clinical seizures before treatment × 100%.

#### EEG result determination criteria (primary outcome)

EEG results were evaluated by the frequency of epileptiform discharges (including spiky waves). A reduction in spiky waves was considered improved, and no change or increase in spiky waves was considered ineffective. The productivity of spike wave (%) = the number of improved cases/the total number of cases × 100%. The degree of change in EEG improvement spike wave values before and after treatment (%) = (the density of spikes before treatment – the density of spikes after treatment)/the density of spikes before treatment × 100%. the density of spikes means number of spikes/3 hours.

#### Emotion-related scales (secondary outcome)

The HAMD score (total score 76) was used to assess depression in both groups before and after treatment: effective: more than 25% of HAMD score reduction, ineffective: less than 25% of HAMD score reduction. The productivity of depression treatment (%) = the number of effective cases /the total number of cases × 100% [21]. The degree of change in HAMD score values before and after treatment (%) = (the total HAMD score before treatment – the total HAMD score after treatment)/the total HAMD score before treatment × 100%.

#### Cognitive function scales (secondary outcome)

The MMSE score (total score of 30) was used to assess the cognitive function of the two groups of patients before and after treatment, and the cognitive ability and score were positively correlated. The degree of change in MMSE score values before and after treatment (%) = (the total MMSE score before treatment – the total MMSE score after treatment (the total MMSE score before treatment – the total MMSE score after treatment)/the total MMSE score before treatment × 100%.

#### Quality of life (secondary outcome)

Before and after 1 month of treatment, Quality of life was evaluated based on the Generic Quality of Life Inventory-74 (GQOL-74), Cronbach’s α = 0.915, including four dimensions of social function and psychological function, with a total of 100 points for each dimension. The higher the score, the better the quality of life [22]. 

#### Adverse reactions (secondary outcome)

We observed whether adverse reactions occurred during treatment for comparison.

### Statistical methods

SPSS 13.0 statistical software was applied for data processing. The measurement data were recorded as x ± s, and the count data were recorded as frequencies or percentages (%). We use Levene’s test for equality of variances to evaluate the homogeneity of variances and Shapiro-Wilk’s Tests of Normality to evaluate normality. The analysis of variance test (F test) was used for comparison between two groups for the measures; If the data is distributed, we used the Welch test to measure. The count data were expressed as [n (%)], and the χ2 test was used for comparison between the two groups. Fish’s exact test was used when the sample size *n* < 40, the theoretical frequency T < 1 in any frame, or the theoretical frequency T < 5 in more than 20% of the frames. The correlations between whether to perform repetitive rTMS therapy and possible relevant measurement factors were analyzed by Spearman analysis. A 0.05 test level was used, and *P* < 0.05 suggested that the differences were statistically significant.

## Results

### Comparison of the efficacy of patients in the treated group and the control group

There was no difference in general information between the treated group and the control group (*P* > 0.05), as shown in Table 1. There were 41 generalized onset cases and 12 focal onset cases in the Treated group, while 49 generalized onset cases and 19 focal onset cases were in the Control group. During the study period, all patients’ TDM of Lvetiracetam was within the normal range, so the drug dosage was not adjusted.

**Table 1:** Comparison of general information between the treated group and the control group

	Treated	Control	χ2/F	P
Gender (M/F)	31/22	34/34	0.864	0.353
Age	67.68 ± 13.48 13.47758 13.47758	64.56 ± 12.81	1.688	0.196
NIHSS score	7.17 ± 9.00	4.79 ± 5.52	3.197	0.076
Hypertension (N/Y)	9/44	13/55	0.091	0.762
Diabetes(N/Y)	38/15	49/19	0.002	0.965
Atrial fibrillation (N/Y)	52/1	62/6	2.629	0.105
Hyperlipidemia (N/Y)	41/12	48/20	0.702	0.402
Coronary heart disease (N/Y)	44/9	57/11	0.012	0.906
Family history of epilepsy (N/Y)	51/2	65/3	0.031	0.861
Smoking History (N/Y)	43/10	48/20	1.776	0.183
Drinking History (N/Y)	48/5	61/7	0.025	0.875

The distribution of different lesion sites in the treated and the control groups was distributed side by side. There was no difference in general information between the treated group and the control group on the location of the lesion (*P* > 0.05), as shown in Table 2.

**Table 2:** Number of cases with different lesion sites in the treated group and the control group (unit: pcs)

Lesion site	Treated	Control	Total	χ2	P
frontal lobe	16	19	35	2.375	0.667
temporal lobe	16	15	31		
parietal lobe	10	12	22		
occipital lobe	7	15	22		
insular lobe	4	7	11		
Total	53	68	121		

The number of EEG spiky waves and the frequency of clinical seizures were significantly reduced in the rTMS group compared with the control group before and after treatment, and the difference between the groups was statistically significant (*P* < 0.05). It is suggested that both methods (single antiepileptic drug therapy or drug combined with rTMS therapy) can reduce epileptiform discharges and clinical seizures. The difference between the MMSE and HAMD scores before and after rTMS treatment was statistically significant (*P* < 0.05), suggesting that cognitive function and depressive symptoms were significantly improved after the drug combined with rTMS therapy. In the control group, the total HAMD score was significantly better before and after treatment, the difference between groups was statistically significant (*P* < 0.05), and the depressive symptoms were significantly better. It is suggested that treatment with antiepileptic drugs alone can significantly improve depressive symptoms. There was no statistically significant difference in the total MMSE scores between the control group before and after treatment (*P* > 0.05). This suggests that treatment with antiepileptic drugs alone cannot improve cognitive impairment. (Table 3) (See Tables 1, 2 and 3 in the Supplementary Material for detailed data).

**Table 3:** Changes before and after treatment in the treated group and control group

	Treated				Control				
	Before	After	χ2/F	P	Before	After	χ2/F	P	
the density of spikes (Unit: times)	23 ± 6	9 ± 5	199.355	0*	22 ± 6	12 ± 7	81.989	0*	
the frequency of clinical seizures (Unit: pcs/month)	8 ± 2	2 ± 1	355.420	0*	8 ± 2	4 ± 2	164.667	0*	
MMSE score	22.34 ± 1.63	24.28 ± 2.88	26.044	0*	22.31 ± 1.62	21.38 ± 4.30	2.768	0.098	
HAMD score	25.15 ± 6.63	14.70 ± 5.47	78.296	0*	24.35 ± 6.81	17.60 ± 6.03	37.444	0*	

* *P* < 0.05

**Fig. 2:**
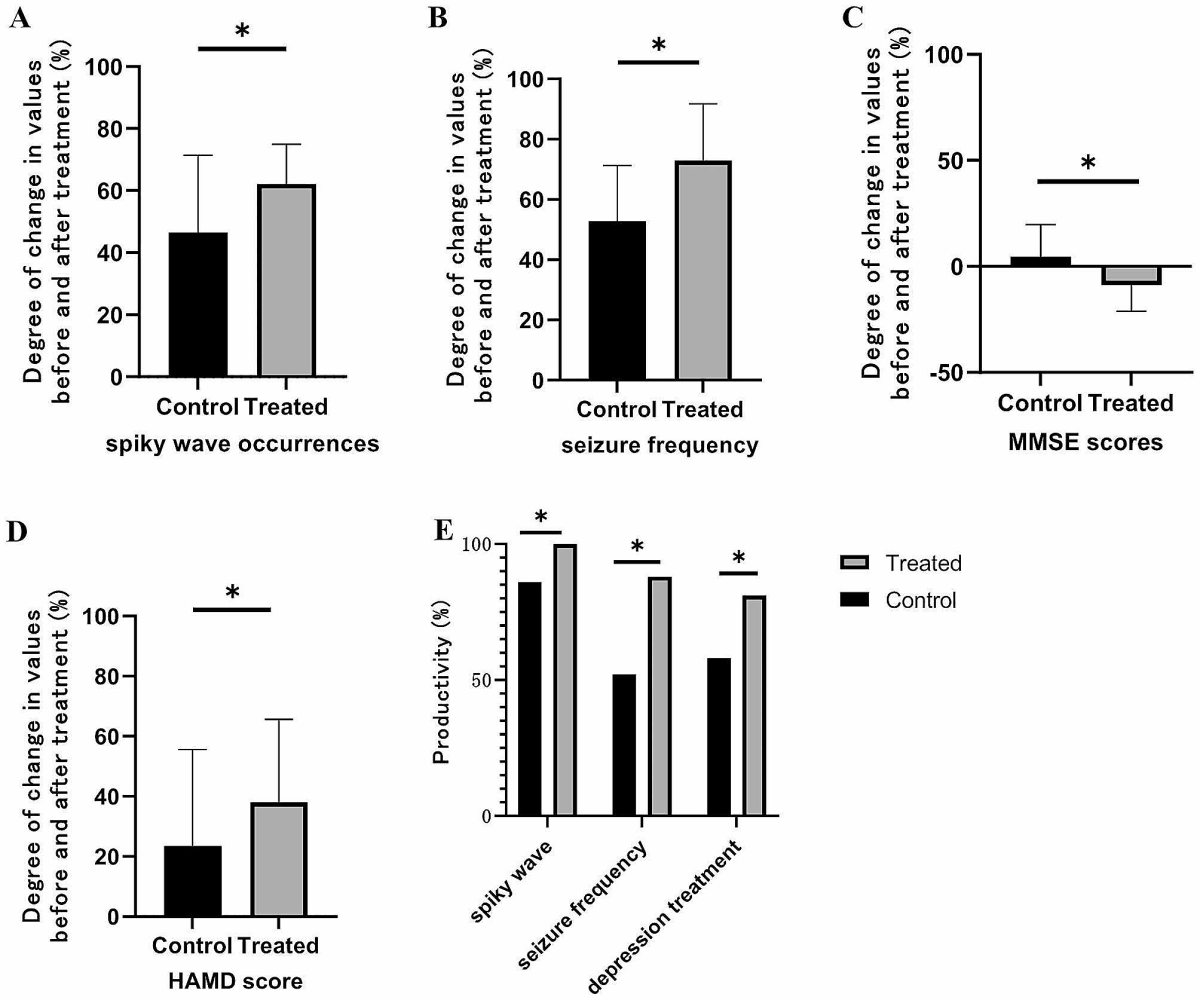
Improvement of the treated group and control group before and after treatment. **A**: the degree of change in seizure frequency in the treated group and control group after treatment; **B**: the degree of change in spiky wave values in the treated group and control group after treatment; **C**: the degree of change in HAMD score in the treated group and control group after treatment; **D**: the degree of change in MMSE score in the treated group and control group after treatment; **E**: the productivity of seizure frequency, spiky wave and depression treatment in the treated group and control group after treatment. * *P* < 0.05

In the treated group, the degree of change in seizure frequency, the degree of change in spiky wave values, and the degree of change in HAMD and MMSE score value were significantly improved after treatment, and the difference between the groups was statistically significant (*P* < 0.05). It is suggested that compared with drug therapy alone, the combination of antiepileptic drugs and rTMS can effectively reduce epileptiform discharge and clinical seizures and improve depression. In the treated group, The productivity of seizure frequency, spiky wave and depression treatment were significantly better than those in the control group after treatment, and the difference between groups was statistically significant (*P* < 0.05). It is suggested that compared with drug therapy alone, antiepileptic drugs combined with rTMS can significantly reduce epileptiform discharge and clinical seizures and significantly improve cognitive impairment and depression. (Fig. 2) (See Tables 4, 5, 6 and 7 in the Supplementary Material for detailed data).

### Comparison of the efficacy of rTMS therapy for patients with cerebral infarction at different sites

**Fig. 3:**
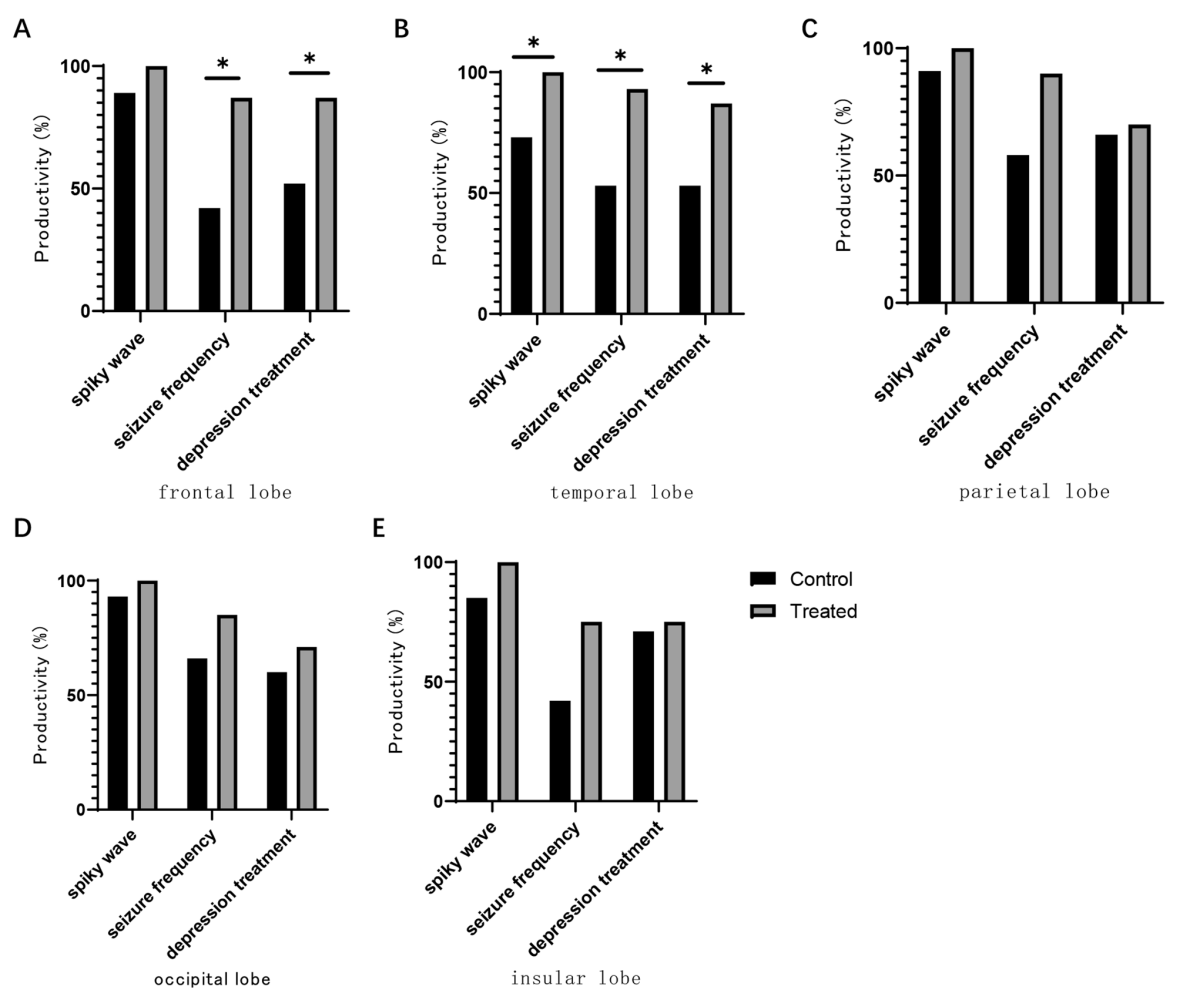
Treatment efficiency of the treated group and control group after treatment of different lesions. **A**: for the patients with lesions in the frontal lobe, the productivity of seizure frequency, spiky wave and depression treatment in the treated group and control group after treatment; **B**: for the patients with lesions in the temporal lobe, the productivity of seizure frequency, spiky wave and depression treatment in the treated group and control group after treatment; **C**: for the patients with lesions in the parietal lobe, the productivity of seizure frequency, spiky wave and depression treatment in the treated group and control group after treatment; **D**: for the patients with lesions in the occipital lobe, the productivity of seizure frequency, spiky wave and depression treatment in the treated group and control group after treatment; **E**: for the patients with lesions in the insular lobe, the productivity of seizure frequency, spiky wave and depression treatment in the treated group and control group after treatment. * *P* < 0.05

In the treated group, the EEG of patients with lesions in the temporal lobe suggested that the productivity was significantly better than that of the control group, and the difference between the two groups was statistically significant (*P* < 0.05); the EEG of patients with lesions in the frontal, parietal, occipital and insular lobes suggested that the difference between the productivity and that of the control group was not statistically significant (*P* > 0.05). It is suggested that the combination of antiepileptic drugs and rTMS can effectively reduce epileptiform discharge in patients with temporal lobe lesions compared with drug therapy alone. For clinical seizure frequency, patients with lesions in the frontal and temporal lobes showed a statistically significant difference in productivity compared with the control group (*P* < 0.05); patients with lesions in the parietal, occipital and insular lobes showed no statistically significant difference in productivity compared with the control group (*P* > 0.05). It is suggested that compared with drug therapy alone, the combination of antiepileptic drugs and rTMS can effectively reduce clinical seizures in patients with frontal and temporal lobe lesions. Patients with lesions in the frontal and temporal lobes had significantly better productivity of depression treatment than the control group, and the difference was statistically significant (*P* < 0.05); patients with lesions in the parietal, occipital and insular lobes had no statistically significant difference in treatment productivity compared with the control group (*P* > 0.05). It is suggested that compared with drug therapy alone, the combination of antiepileptic drugs and rTMS can effectively improve depression in patients with frontal and temporal lobe lesions. (Fig. 3) (See Table 8, and 9 in Supplementary Material for detailed data) In addition, the productivity of clinical seizure frequency for all cases in the treated group was decreased. So rTMS is effective for all types of seizures.

**Fig. 4:**
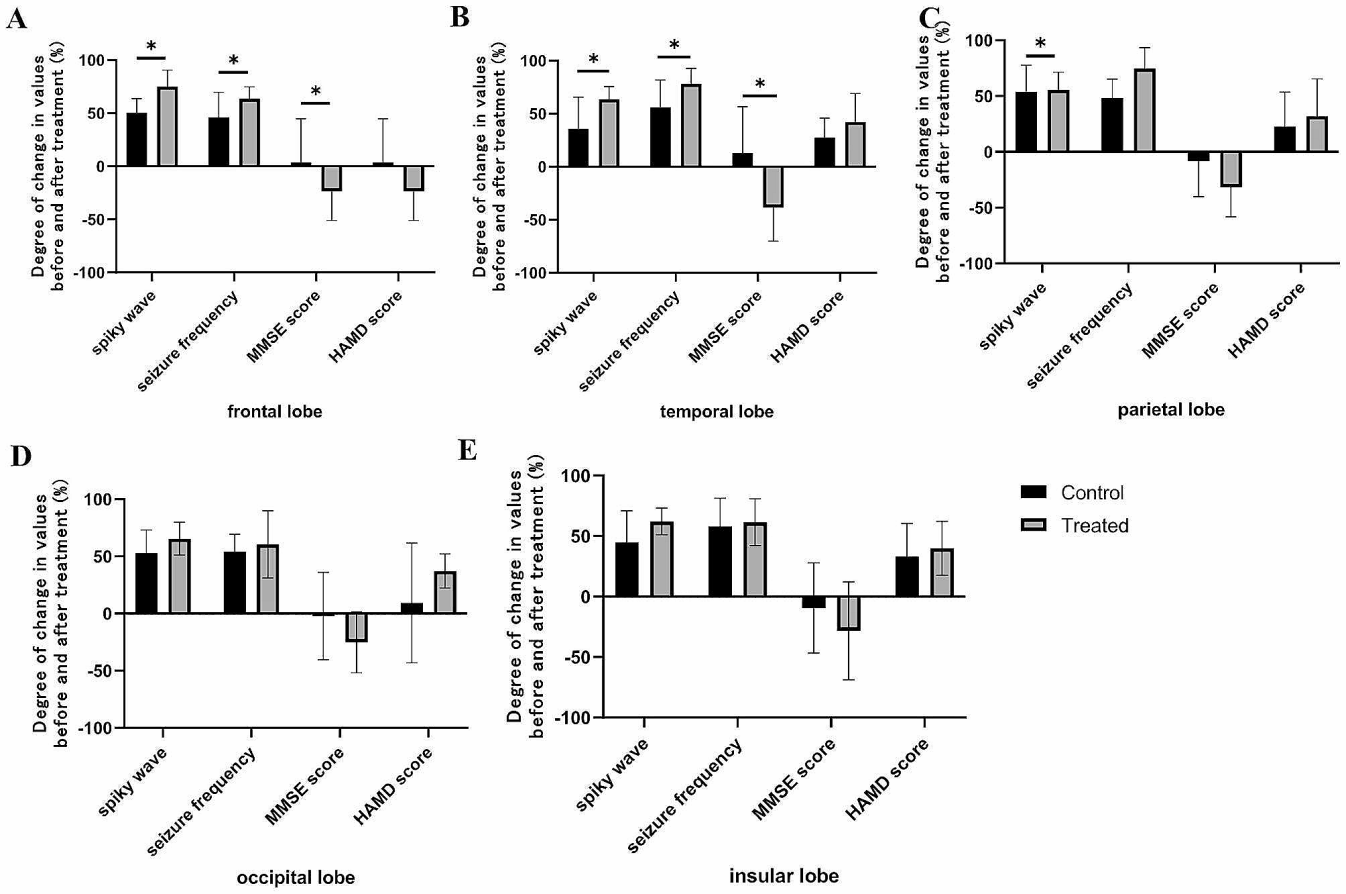
Degree of change between the treated group and the control group after treatment of different lesions. **A**: for the patients with lesions in the frontal lobe, the degree of change in seizure frequency, spiky wave values, HAMD and MMSE score in the treated group and control group after treatment; **B**: for the patients with lesions in the temporal lobe, the degree of change in seizure frequency, spiky wave values, HAMD and MMSE score in the treated group and control group after treatment; **C**: for the patients with lesions in the parietal lobe, the degree of change in seizure frequency, spiky wave values, HAMD and MMSE score in the treated group and control group after treatment; **D**: for the patients with lesions in the occipital lobe, the degree of change in seizure frequency, spiky wave values, HAMD and MMSE score in the treated group and control group after treatment; **E**: for the patients with lesions in the insular lobe, the degree of change in seizure frequency, spiky wave values, HAMD and MMSE score in the treated group and control group after treatment. *P < 0.05

In the treated group, the EEG of patients with lesions in the frontal, temporal and parietal lobes showed a statistically significant difference in the degree of change of spiky wave issuance compared with the control group (*P* < 0.05); for patients with lesions in the parietal, occipital and insular lobes, the difference between the EEG between the groups was not statistically significant (*P* > 0.05). It is suggested that compared with drug treatment alone, in patients with lesions in the frontal lobe, temporal lobe and parietal lobe, the combination of antiepileptic drugs and rTMS can significantly reduce epileptiform discharge in patients with frontal, temporal and parietal lobes lesions. The degree of change in seizure frequency in patients with lesions in the frontal, temporal and parietal lobes was significantly better than that in the control group, and the difference between the groups was statistically significant (*P* < 0.05); for patients with lesions in the occipital and insular lobes, the difference between the groups was not statistically significant (*P* > 0.05). It is suggested that compared with drug therapy alone, the combination of antiepileptic drugs and rTMS can significantly reduce clinical seizures in patients with frontal and temporal lobe lesions. In patients with lesions in the frontal and temporal lobes, the degree of change in MMSE scores after treatment was significantly better than that in the control group, and the difference between the groups was statistically significant (*P* < 0.05); in patients with lesions in the parietal, occipital and insular lobes, the difference between the degree of change in MMS scores after treatment and the control group was not statistically significant (*P* > 0.05). It is suggested that compared with drug therapy alone, the combination of antiepileptic drugs and rTMS can significantly improve cognitive scale scores in patients with frontal and temporal lobe lesions. Patients with lesions in the frontal lobe showed significantly better HAMD scale scores than the control group after treatment, and the difference between the groups was statistically significant (*P* < 0.05); patients with lesions in the temporal, parietal, occipital, and insular lobes showed no statistically significant difference between the groups and the control group after treatment (*P* > 0.05). It is suggested that in patients with different lesions, there is no significant difference between anti-epileptic drugs combined with rTMS therapy and drug therapy alone for depression scale score reduction. (Fig. 4) (See Table 8, and 9 in the Supplementary Material for detailed data).

### Quality of life

GQOL-74 scores in the social function and psychological function were higher than those in the control group. There was a statistically significant difference in the GQOL-74 scores between the two groups (*P* > 0.05) (Table 4). It is suggested that anti-epileptic drugs combined with rTMS therapy can improve patients’ quality of life better than drug therapy alone.

**Table 4:** Comparison of GQOL-74 scores between the treated group and the control group before and after treatment

	n	social function		psychological function	
		Before	After	Before	After
Treated	53	67.06 ± 5.67	81.32 ± 0.661^a^	63.49 ± 5.84	81.41 ± 0.662^a^
Control	68	67.60 ± 6.27	77.75 ± 0.583^a^	64.09 ± 6.38	78.19 ± 0.585^a^
F		16.36^a^		13.283^a^	
P		0^a*^		0^a*^	

* *P* < 0.05, a. from ANCOVA.

### Correlation analysis

The population did not conform to a normal distribution, so we analyzed the correlations between whether to perform repetitive rTMS therapy and possible relevant measurement factors with Spearman analysis.

There are significant (*P* < 0.05) correlativity between presence or absence of rTMS and the degree of change in seizure frequency, the degree of change in spiky wave values, the productivity of depression treatment, and the degree of change in MMSE score value (Table 5). Correlation Coefficient: the degree of change in seizure frequency > the degree of change in MMSE score value > the degree of change in spiky wave values > the productivity of depression treatment.

**Table 5:** Spearman analysis of whether to perform repetitive rTMS therapy and clinical seizures, EEG, improvement in MMSE scores, and effectiveness of depression treatment

	Correlation Coefficient	P	N
The degree of change in spiky wave value	0.348279	0*	121
The degree of change in seizure frequency	0.512609	0*	121
The productivity of depression treatment	0.238469	0.008*	121
The degree of change in MMSE score value	-0.45406	0*	121

* *P* < 0.05

### Safety check

There was no statistically significant difference in the incidence of adverse reactions between the treated group and the control group (*P* > 0.05); rTMS treatment did not increase adverse reactions (Table 6).

**Table 6:** Comparison of the incidence of adverse reactions in the treated group and the control group

	n	No adverse reactions	Dizziness	Headache	Nausea	drowsiness	Incidence of adverse reactions (%)	χ2	P
Treated	53	45	2	3	1	1	13.46	0.001	0.971
Control	68	59	3	2	1	3	13.24		

## Discussion

### Efficacy for epilepsy

This study shows that treatment with antiepileptic drugs alone or drugs combined with rTMS can reduce epileptiform discharges and clinical seizures (Table 3). Levetiracetam is a new antiepileptic drug. It can combine with synaptic vesicle protein 2 A (SV2A) in the brain; it can prevent hippocampal CA1 pyramidal neurons from activating N-type calcium channels through high voltage; and it can reduce the content of γ-aminobutyric acid (GABA) receptor in the cerebral cortex, thus enhancing the inhibitory effect of GABA on the neuronal circuit and finally exerting an antiepileptic effect [23]. The excitability of repeated cortical discharges is related to the plasticity of the cortex, which means that the cortex cannot maintain a stable level of excitability and has inhibitory control defects. [24, 25] rTMS can reduce clinical seizures by suppressing cortical excitability that can inhibit epileptic activity [12]. Meanwhile, rTMS can induce ribosomal protein S6 phosphorylation in large numbers of neurons and structural alterations of synaptic plasticity to increase cortical plasticity. [26, 27] Both can control epilepsy through different mechanisms.

However, compared with drug therapy alone, drug therapy combined with rTMS can significantly improve the clinical seizures and epileptiform discharges of the brain after stroke (Fig. 2), especially for patients with lesions in the frontal and temporal lobes (Figs. 3 and 4). It may be that antiepileptic drugs and rTMS therapy have different antiepileptic mechanisms and can also play a synergistic role. Inflammatory reactions and abnormal immune function play an important role in the occurrence and development of epilepsy, while tumor necrosis factor-α (TNF-α) is an important inflammatory cytokine that can promote the activation of B, T and mononuclear macrophages, promote the expression of other inflammatory cytokines such as interleukin-6 and interleukin-2, and accelerate the secretion of human leukocyte class II antigen by antigen-presenting cells, thus participating in the occurrence and progression of epilepsy. Intercellular adhesion molecule-1 (ICAM-1) is an immunoglobulin that can promote the reorganization of the endothelial cytoskeleton by combining with its receptor, accelerate the adhesion of vascular endothelial cells and leukocytes, trigger cascade reactions, cause rearrangement of leukocyte cytoskeleton and activation of leukocytes, promote the occurrence and progression of inflammation, patient’s condition. Some studies have shown that levetiracetam combined with rTMS can reduce serum ICAM-1 and TNF-α levels and alleviate the inflammatory reaction of the body [23]. This suggests that levetiracetam and rTMS may have the same mechanism to control clinical seizures. Therefore, drugs combined with rTMS can help to control epilepsy faster. The decrease in neuronal and glial density and size at the site of the lesion (e.g., frontal lobe) after stroke onset and the hyperactivity of the hypothalamic‒pituitary‒adrenal axis (HPAA) lead to increased blood cortisol concentrations and excess synaptic glutamate, causing cortical hyperactivity, which can increase clinical seizures [28]. The “figure-eight” coil consists of two separate circular coils opposite each other, with the strongest combined magnetic field at the point of contact between the coils [10], and can therefore be used for targeted treatment of local epileptogenic foci. We placed figure-eight coils on epileptogenic foci for inhibitory stimulation to control clinical seizures, and this result is the same as Wang’s research [29]. The results of this experiment show that the combination of drugs and rTMS can also significantly improve the epileptiform discharge of patients with parietal lobe lesions after stroke compared with drug treatment alone (Fig. 4). There has been evidence that there is a close relationship between the frontal lobe and the parietal lobe. Local hyperexcitation of the parietal cortex can lead to increased connectivity with the frontal lobe, which can cause disease progression [30].

### Effects on depressive status

This study shows that simple antiepileptic drug therapy and drug therapy combined with rTMS can significantly improve depressive symptoms (Table 3). Compared with drug therapy alone, antiepileptic drugs combined with rTMS can significantly reduce epileptiform discharges and clinical seizures, significantly improve depression (Fig. 2), are effective for patients with lesions in the frontal lobe and temporal lobe, and are more effective for patients with lesions in the frontal lobe (Figs. 3 and 4). It may be the same as the above mechanism of epilepsy in depressive states. Plasma cortisol concentrations are elevated due to a decrease in glial or neuronal cell density and size in the cingulate gyrus, layers II, III and IV of the anastomotic orbitofrontal cortex, and layers V and VI of the caudal orbitofrontal cortex. They can also occur due to a decrease in neuronal and glial density and size in all cortical layers of the dorsolateral prefrontal cortex. High cortisol concentrations can affect cortical excitability by influencing neurotransmitters, including glutamate, serotonin and GABA. Meanwhile, a study brings out a decrease of connectivity between the left t dorsolateral prefrontal cortex and both the cingulate/medial frontal cortex and bilateral medial temporal limbic area can lead to remote temporal hypoperfusion and play an antidepressant role [31]. In turn, neurotransmitters are involved in the common pathogenic mechanisms of depressive states and epilepsy. For example, in patients with temporal lobe epilepsy, hyperactivity of HPAA leads to elevated blood cortisol concentrations, which is a feature of depressive states. The reduction in glial cell density and function associated with high cortisol concentrations leads to an excess of synaptic glutamate, exacerbating the depressive state [28]. This increases the connections between other cortical layers, enhancing cortical excitability and contributing to improved cognitive function, thereby improving mood [12]. Furthermore, Triggs et al. showed that dorsolateral prefrontal rTMS in depressed patients is associated with distal changes in cortical excitability, such as changes in motor-evoked potential thresholds, thus demonstrating that stimulation of the prefrontal cortex leads to a decrease in cortical excitability in distal areas. According to this concept, the modulation of the prefrontal cortex by rTMS may also modulate distal cortical or subcortical epileptic foci [12]. Therefore, reducing the activity of epileptic foci may increase frontal cortical excitability and thus improve depression.

### Effects on cognitive function

The results of this experiment showed that treatment with antiepileptic drugs alone could not improve cognitive impairment (Table 3).In contrast, drugs combined with rTMS can effectively improve cognitive impairment, especially for patients with lesions in the frontal and temporal lobes (Table 3; Fig. 4). The prefrontal lobe is associated with memory, judgment, manipulation, and analysis. These are important brain regions related to intelligence. For temporal lobe epileptic patients who receive inhibitory rTMS from the temporal lobe, the inhibitory temporal to prefrontal input is reduced, resulting in a secondary increase in frontal lobe activity. This increases the connections between other cortical layers, enhancing cortical excitability and contributing to improved cognitive function [12]. However, the excessive excitability of the frontal lobe is also related to the decline in cognitive function. The balance of neuronal excitation/inhibition depends on the change in Aβ. The increase in soluble Aβ concentration can increase the excitability of the prefrontal lobe, which is related to the occurrence of more serious cognitive impairment, especially in the later stage of the disease [32]. Therefore, patients with frontal lobe epilepsy can improve their cognitive function after receiving inhibitory rTMS treatment. Cognitive impairment often occurs in the early stage of depression. Even if depression is relieved, cognitive impairment will continue. Moreover, the greater the number of depressive episodes, the more serious the cognitive impairment. Furthermore, cognitive impairments may increase the risk of depression relapse [33]. The persistence and aggravation of cognitive impairment and depression is a vicious cycle. Previous studies have shown that for patients with early depression and cognitive impairment, the degree of cognitive impairment can be reversed by treatment, and rTMS is an effective treatment method [33, 34]. Therefore, rTMS can improve depression and reduce the further development of cognitive impairment. Mutual improvement of cognitive impairment and depression is a virtuous cycle and promotes the recovery of patients.

### Correlation

Correlation analysis further verified that clinical seizures, EEG, MMSE score improvement and depression treatment effectiveness were correlated with the presence or absence of rTMS. Therefore, the application of rTMS in the treatment of traditional antiepileptic drugs is a good way to treat epilepsy with cognitive impairment and depression.

The degree of change in seizure frequency has the greatest correlation with rTMS treatment, suggesting that rTMS has the best effect on improving clinical seizures. We also found similar results in other studies [35]- for the treatment of epilepsy, rTMS significantly improved the seizure frequency compared with the degree of change in spiky wave values. But at present, the relevant mechanism is not clear, and there are few studies on the difference between epileptic seizures and EEG improvement.

The correlation between the degree of change in MMSE score value and rTMS treatment is second only to the degree of change in severity frequency, suggesting that rTMS has the best effect in treating post-stroke epilepsy, followed by cognitive impairment, and then depression. At present, most studies [36] only show that rTMS can improve cognitive impairment and depression, but there is no study to explain the difference between cognitive impairment and depression, especially in patients with epilepsy after stroke. This may require further large-scale research to verify whether the experimental results are consistent; At the same time, we should further study the mechanism of the difference of curative effect between epilepsy, cognitive impairment and depression. This may be our next research direction.

### Safety

The results of this experiment suggest that rTMS treatment does not increase adverse effects. Since the effects of rTMS are focal, it is safe for other distant cortical functions. In addition, rTMS can be performed without the potential side effects of antiepileptic drugs. Finally, although other side effects of rTMS, such as transient headache and neck pain, have been reported, these effects are generally mild and last only for a relatively short period of time [12].

### Limitations

The present study has the limitation of failing to further investigate the clinical manifestations of different cognitive impairment and depressive states (e.g., memory impairment in patients with temporal lobe epilepsy and executive function in patients with frontal lobe epilepsy [21]) that occur with different lesions treated with repeated rTMS. This paper is a single-center, retrospective study with a small sample size and possible experimental error, and further larger sample, multicenter, prospective, randomized controlled trials are still needed to validate the results.

## Conclusion

Based on our findings, we propose that rTMS can improve the cognitive impairment and depressive status of comorbid epileptic patients by reducing clinical seizures. Therefore, we suggest that low-frequency rTMS can be used as an adjunct treatment to antiepileptic drugs, providing some treatment ideas and references for patients with epilepsy with cognitive impairment and depression.

### Electronic supplementary material

Below is the link to the electronic supplementary material.

Supplementary Material 1

Supplementary Material 2

## Data Availability

The datasets used and/or analyzed during the current study available from the corresponding author on reasonable request.

## Code Availability

The software application used in this study was SPSS for Windows, version 13.0 (IBM Corp., Armonk, N.Y., USA).
