# Supplementary material for: Efficacy of rTMS for poststroke epilepsy and its effects on patients’ cognitive function and depressive status
Source: BMC Neurol. 2024 Jan 12;24:25. doi: 10.1186/s12883-024-03531-4 (PMC10785375; doi:10.1186/s12883-024-03531-4)
Supplement: Supplementary file 1 — Supplementary Material 1 [file 12883_2024_3531_MOESM1_ESM.docx]

Supplementary Material

Efficacy of transcranial magnetic stimulation for post-stroke epilepsy and its effects on patients' cognitive function and depressive status

Table 1 EEG changes before and after treatment in the TMS treatment group and control group

| group | Number of spiky wave occurrences (Unit: times) | | Sig.  (Tests of Normality) | Sig.  (Test of Homogeneity of Variances) | F | P |
| --- | --- | --- | --- | --- | --- | --- |
|  | Before | After |  |  |  |  |
| Treated | 22.94 | 8.87 | 0.136 | 0.130 | 199.355 | 0^*^ |
| Control | 22.40 | 12.10 | 0 | 0.261 | 81.989 | 0^*^ |

* P<0.05

Table 2 Changes in clinical seizures before and after treatment in the TMS treatment group and the control group

| Group | seizure frequency  (Unit: pcs/month) | | Sig.  (Tests of Normality) | Sig.  (Test of Homogeneity of Variances) | F | P |
| --- | --- | --- | --- | --- | --- | --- |
|  | Before | After |  |  |  |  |
| Treated | 8±2 | 2±1 | 0.000 | 0.094 | 355.420 | 0.000* |
| Control | 8±2 | 4±2 | 0.000 | 0.395 | 164.667 | 0.000* |

* P<0.05

Table 3 Changes in MMSE and HAMD scores before and after treatment in the TMS treatment group and control group

|  |  | MMSE score | HAMD score |
| --- | --- | --- | --- |
| Treated | Before | 22.34±1.63 | 25.15±6.63 |
|  | After | 24.28±2.88 | 14.70±5.47 |
|  | Sig. (Tests of Normality) | 0.004 | 0.000 |
|  | Sig.(Test of Homogeneity of Variances) | 0.000 | 0.007 |
|  | F | 18.305 | 78.296 |
|  | P | 0* | 0* |
| Control | Before | 22.31±1.62 | 24.35±6.81 |
|  | After | 21.38±4.30 | 17.60±6.03 |
|  | Sig. (Tests of Normality) | 0.006541 | 0 |
|  | Sig.(Test of Homogeneity of Variances) | 0.000 | 0.073 |
|  | F | 2.768 | 37.444 |
|  | P | 0.098 | 0.000^*^ |

* P<0.05

Table 4 EEG improvement after treatment in the TMS treatment group and the control group

| group | Effective | Invalid | Productivity (%) | Degree of change (%) |
| --- | --- | --- | --- | --- |
| Treated | 53 | 0 | 100 | 62.226 |
| Control | 59 | 9 | 86.765 | 46.536 |
| Sig. (Tests of Normality) | - | - | - | 0 |
| Sig.(Test of Homogeneity of Variances) | - | - | - | 0 |
| χ2/F | - | - | 7.578 | 17.425 |
| P | - | - | 0.006^*^ | 0.000^*^ |

* P<0.05

Table 5 Clinical seizure improvement in the TMS treatment group and the control group

| group | Effective | Invalid | Productivity (%) | Degree of change (%) |
| --- | --- | --- | --- | --- |
| Treated | 47 | 6 | 88.679 | 72.919 |
| Control | 36 | 32 | 52.941 | 52.792 |
| Sig. (Tests of Normality) | - | - | - | 0 |
| Sig.(Test of Homogeneity of Variances) | - | - | - | 0.655 |
| χ2/F | - | - | 17.659 | 40.083^a^ |
| P | - | - | 0.000^*^ | 0.000^a*^ |

* P<0.05, a. from ANCOVA.

Table 6 Degree of change in MMSE and HAMD scores in the TMS treatment group and control group

| group | MMSE score | HAMD score |
| --- | --- | --- |
| Treated | 0.05±0.15 | 38.01±0.28 |
| Control | -0.09±0.12 | 23.47±0.32 |
| Sig. (Tests of Normality) | 0.007 | 0.000 |
| Sig.(Test of Homogeneity of Variances) | 0.087 | 0.381 |
| F | 25.169^a^ | 8.658^a^ |
| P | 0.000^a*^ | 0.004^a*^ |

* P<0.05, a. from ANCOVA.

Table 7 Effective posttreatment depression treatment in the TMS treatment group and the control group

| Group | Effective | Invalid | Productivity (%) | χ2 | P |
| --- | --- | --- | --- | --- | --- |
| Treated | 43 | 10 | 81.132 | 6.881 | 0.009* |
| Control | 40 | 28 | 58.824 |  |  |

* P<0.05

Table 8 Clinical seizures and EEG improvement in the TMS treatment group and control group after treatment of different lesions

| Lesion site | | The change of epileptic seizure (%) | The Productivity of epileptic seizure (%) | The change of epileptic discharge (%) | The Productivity of epileptic discharge (%) |
| --- | --- | --- | --- | --- | --- |
| frontal lobe | Control | 50.20±13.55 | 42.105 | 46.11±11.21 | 89.47 |
|  | Treated | 75.10±15.50 | 87.50 | 63.59±23.58 | 100.00 |
|  | Sig. (Tests of Normality) | 0.000 | - | 0.000 | - |
|  | Sig.(Test of Homogeneity of Variances) | 0.433 | - | 0.031 | - |
|  | χ2/F | 30.935^a^ | 7.666 | 6.862^a^ | 1.786 |
|  | P | 0.000^a*^ | 0.006* | 0.013^a*^ | 0.200 |
| temporal lobe | Control | 55.93±25.78 | 53.33 | 35.53±29.98 | 73.33 |
|  | Treated | 78.07±14.64 | 93.75 | 63.66±11.99 | 100.00 |
|  | Sig. (Tests of Normality) | 0.007 | - | 0.001 | - |
|  | Sig.(Test of Homogeneity of Variances) | 0.040 | - | 0.001 | - |
|  | χ2/F | 8.797 | 14.564^a^ | 12.078^a^ | 4.899 |
|  | P | 0.006* | 0.001^a*^ | 0.002^a*^ | 0.027^*^ |
| parietal lobe | Control | 48.35±16.85 | 58.33 | 53.99±23.61 | 91.67 |
|  | Treated | 74.53±18.96 | 90.00 | 55.53±15.81 | 100.00 |
|  | Sig. (Tests of Normality) | 0.067 | - | 0.003 | - |
|  | Sig.(Test of Homogeneity of Variances) | 0.433 | - | 0.560 | - |
|  | χ2/F | 9.604^a^ | 2.758 | 0.031 | 0.873 |
|  | P | 0.006^a*^ | 0.097 | 0.862 | 0.350 |
| occipital lobe | Control | 54.06±15.12 | 66.67 | 52.96±20.11 | 93.33 |
|  | Treated | 60.44±29.40 | 85.71 | 65.49±14.28 | 100.00 |
|  | Sig. (Tests of Normality) | 0.083 | - | 0.004 | - |
|  | Sig.(Test of Homogeneity of Variances) | 0.130 | - | 0.550 | - |
|  | χ2/F | 0.462 | 0.873 | 2.176 | 0.489 |
|  | P | 0.504 | 0.350 | 0.156 | 0.484 |
| insular lobe | Control | 58.005±23.32 | 42.86 | 44.72±22.40 | 85.71 |
|  | Treated | 61.39±19.36 | 75 | 62.05±11.03 | 100.00 |
|  | Sig. (Tests of Normality) | 0.036 | - | 0.202 | - |
|  | Sig.(Test of Homogeneity of Variances) | 0.778 | - | 0.168 | - |
|  | χ2/F | 3.333^a^ | 1.061 | 1.538 | 0.629 |
|  | P | 0.105^a^ | 0.303 | 0.246 | 0.428 |

* P<0.05, a. from ANCOVA.

Table 9 The improvement of MMSE and HAMD scale scores in the TMS treatment group and the control group after treatment of different lesions

| Lesion site | | MMSE score | HAMD score | The Productivity of depression (%) |
| --- | --- | --- | --- | --- |
| frontal lobe | Control | 0.03±0.13 | 37.47±19.28 | 52.63 |
|  | Treated | -0.15±0.10 | 37.47±31.80 | 87.50 |
|  | Sig. (Tests of Normality) | 0.408 | 0.002 | - |
|  | Sig.(Test of Homogeneity of Variances) | 0.381 | 0.642 | - |
|  | χ2/F | 20.023^a^ | 1.491^a^ | 4.900 |
|  | P | 0^a*^ | 0.231^a^ | 0.027^*^ |
| temporal lobe | Control | 0.09±0.16 | 27.42±18.37 | 53.33 |
|  | Treated | -0.13±0.09 | 42.26±26.81 | 87.50 |
|  | Sig. (Tests of Normality) | 0.0154 | 0.114 | - |
|  | Sig.(Test of Homogeneity of Variances) | 0.006 | 0.202 | - |
|  | χ2/F | 20.017^a^ | 3.190 | 4.386 |
|  | P | 0^a*^ | 0.085 | 0.036^*^ |
| parietal lobe | Control | 0.03±0.12 | 22.72±33.61 | 70.00 |
|  | Treated | -0.02±0.11 | 31.88±30.87 | 66.67 |
|  | Sig. (Tests of Normality) | 0.106 | 0.017 | - |
|  | Sig.(Test of Homogeneity of Variances) | 0.635 | 0.912 | - |
|  | χ2/F | 1.261^a^ | 0.198^a^ | 0.021^a^ |
|  | P | 0.275^a^ | 0.661^a^ | 0.886^a^ |
| occipital lobe | Control | 0.02±0.19 | 9.23±52.38 | 71.429 |
|  | Treated | 0.02±0.13 | 37.18±14.91 | 60 |
|  | Sig. (Tests of Normality) | 0.231 | 0.000 | - |
|  | Sig.(Test of Homogeneity of Variances) | 0.028 | 0.0144 | - |
|  | χ2/F | 0.001 | 1.876 | 0.004^a^ |
|  | P | 0.971 | 0.186 | 0.951^a^ |
| insular lobe | Control | 0.068±0.157 | 33.28±27.31 | 71.429 |
|  | Treated | -0.06±0.16 | 39.93±22.40 | 75 |
|  | Sig. (Tests of Normality) | 0.118 | 0.673 | - |
|  | Sig.(Test of Homogeneity of Variances) | 0.945 | 0.495 | - |
|  | χ2/F | 2.276^a^ | 0.169 | 0.806^a^ |
|  | P | 0.170^a^ | 0.690 | 0.396^a^ |

* P<0.05, a. from ANCOVA.
